# Macronutrient composition of sea otter diet with respect to recolonization, life history, and season in southern Southeast Alaska

**DOI:** 10.1002/ece3.10042

**Published:** 2023-05-02

**Authors:** Nicole L. LaRoche, Sydney L. King, Emily A. Fergusson, Ginny L. Eckert, Heidi C. Pearson

**Affiliations:** ^1^ College of Fisheries and Ocean Sciences University of Alaska Fairbanks Juneau Alaska USA; ^2^ Department of Natural Sciences University of Alaska Southeast Juneau Alaska USA; ^3^ NOAA National Marine Fisheries Service Alaska Fisheries Science Center, Auke Bay Laboratories Juneau Alaska USA

**Keywords:** ecology, *Enhydra lutris*, foraging, lipid, macronutrient, marine mammal, protein

## Abstract

The sea otter (*Enhydra lutris*) population of Southeast Alaska has been growing at a higher rate than other regions along the Pacific coast. While good for the recovery of this endangered species, rapid population growth of this apex predator can create a human‐wildlife conflict, negatively impacting commercial and subsistence fishing. Previous foraging studies throughout the sea otter range have shown they will reduce invertebrate prey biomass when recolonizing an area. The goal of this study was to examine and quantify the energy content of sea otter diets through direct foraging observations and prey collection. Our study area, Prince of Wales Island in southern Southeast Alaska, exhibits a gradient of sea otter recolonization, thus providing a natural experiment to test diet change in regions with different recolonization histories. Sea otter prey items were collected in three seasons (spring, summer, and winter) to measure caloric value and lipid and protein content. We observed 3523 sea otter dives during the spring and summer. A majority of the sea otter diet consisted of clams. Sea otters in newly recolonized areas had lower diet diversity, higher energetic intake rates (EIR, kcal/min), and prey had higher energy content (kcal/g). Females with pups had the highest diet diversity and the lowest EIR. Sea otter EIR were higher in the fall and winter vs. spring and summer. Sea cucumber energy and lipid content appeared to correspond with times when sea otters consumed the highest proportion of sea cucumbers. These caloric variations are an important component of understanding ecosystem‐level effects sea otters have in the nearshore environment.

## Graphical abstract

Sea otters in southern Southeast Alaska have diets that consist of a majority of clams. Their diets vary slightly in protein and lipid content by season and females with pups had the lowest overall energetic caloric intake rate.

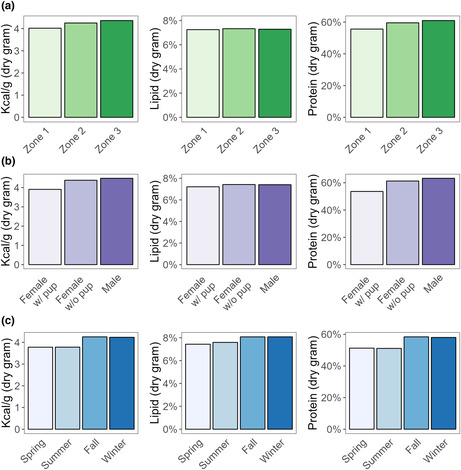

## 1 INTRODUCTION

The nutritional ecology of marine predators is poorly understood compared to terrestrial predators (Machovsky‐Capuska et al., 2018). Classic foraging theory suggests that consumers should target prey that maximizes their net rate of energetic gain (Jenkins & Krebs, 1988; Kleiber, 1961). As energetic intake was the focus, these studies addressed consumers' overall net energetic consumption as a factor in prey selection. However, more recent studies have focused on predators' nutritional needs and how prey choice varies according to macronutrient composition (Machovsky‐Capuska et al., 2018; Oftedal et al., 2007; Vollenweider et al., 2011). Consumers may target lower energy prey for various reasons, including the predator's sex and reproductive status. In marine birds, Machovsky‐Capuska et al. (2018) revealed that male Australasian gannets (*Morus serrator*) consistently foraged for fish with higher protein‐to‐lipid ratios, whereas females foraged for fish with higher lipid‐to‐protein ratios. One potential explanation is the differential dietary response by female and male parents according to the changing needs of growing chicks (Machovsky‐Capuska et al., 2018). In marine mammals, Staedler (2011) revealed that female sea otters (*Enhydra lutris*) will switch their foraging tactics when caring for a pup, which may represent a trade‐off between maximizing potential energy return and meeting pup needs.

Sea otters are a dynamic species in which to study nutritional ecology because of their unique adaptations to the marine environment. Unlike most marine mammals, sea otters do not have blubber to keep them warm. Instead, sea otters use a combination of dense fur for insulation and maintain very high metabolic rates (Liwang et al., 2012; Wright et al., 2021). Various studies estimate sea otters consume anywhere from 19% to 39% of their body weight in food per day to sustain these elevated metabolic costs (Costa, 1982; Davis, 2019). Because of their voracious appetites, sea otters can exert large effects on the nearshore marine ecosystem within relatively short periods (Estes & Palmisano, 1974). These effects are particularly evident in rocky subtidal reefs, where sea otters suppress the grazers, sea urchins (*Strongylocentrotus* spp.), which in turn relieves grazing pressure and allows increased algal abundance (Estes & Duggins, 1995). Increased algal abundance in kelp forests provides fish habitat (Estes & Duggins, 1995) and increases overall species diversity in the system (Steneck et al., 2002).

Historical records show that sea otters once inhabited nearshore ecosystems of the Pacific Ocean from Japan to Baja California. However, by the late 19th Century, there were only 11 remnant populations within their once continuous distribution due to hunting for the lucrative fur markets in Russia and China. In 1911, sea otters were protected from hunting by the International Fur Seal Treaty (Kenyon, 1969). By this time, sea otters were extirpated from Southeast Alaska (Jameson et al., 1982). To restore sea otters to their historical range, the Alaska Department of Fish and Game and the Atomic Energy Commission initiated a translocation program and, in the 1960s, relocated about 400 sea otters from the Aleutian Islands and Prince William Sound to six locations in Southeast Alaska (Burris & McKnight, 1973; Jameson et al., 1982). Since the translocation, sea otters have expanded their range and increased in numbers (Bodkin, 2015). The most recent range‐wide sea otter aerial counts in 2022 estimated that approximately 23,000 sea otters were present in Southeast Alaska (Schutte et al., 2023). The expansion of sea otters from the six translocation sites in Southeast Alaska into unoccupied habitats over time allows for a “space‐for‐time” substitution (Pickett, 1989), in which the longer‐term effects (positive, neutral, and negative) of sea otters on the nearshore ecosystem can be seen in areas of longer occupation.

Prince of Wales Island (POW), along with its neighboring islands, in southern Southeast Alaska has two original translocation release locations. Hoyt (2015) studied sea otter diets around POW for 3 years (2010–2012) focusing on sea otter impacts on commercially important species. Hoyt (2015) found that the number of species consumed by sea otters increased as time since recolonization increased, and sea otters reduced the abundance of commercially important species. The sea cucumber (*Apostichopus californicus*) fishery is an example of a commercial fishery impacted by sea otters. Previous studies showed that sea otter presence was correlated with a decline of sea cucumbers in all regions where sea otters were present for >15 years (Larson et al., 2013). Many sea cucumber fishery regions have been closed due to declining sea cucumber abundance after sea otters have recolonized the regions (Hebert, 2017).

Measuring sea otter energetic intake rate (EIR) is a widely used method to measure changes in diet and to assign quantitative values to sea otter nutritional needs (Dean et al., 2002; Tinker et al., 2008). In Alaska, the Alaska Science Center, a part of the United States Geological Survey (USGS), maintains a database on species‐ and size‐specific energy content for sea otter prey items. Many of these values come from California invertebrate collections and published literature (Oftedal et al., 2007; Tinker, 2004). These values are used to create energetic models and biomass estimates for each sea otter prey species; however, using prey values from other regions could lead to inaccurate consumption estimates for sea otters in Alaska. Similarly, a comprehensive analysis of the biochemical composition of sea otter prey was conducted in varying seasons in California but is absent for Alaskan prey (Oftedal et al., 2007). A preliminary study in Sitka, Alaska, showed that the preferred prey of sea otters (clams) was not the highest in overall caloric content or lipid content when compared to all available prey items (Cartagena da Silva & Matos, 2016). This preliminary work is a driver for further investigation of sea otter diet analysis in Southeast Alaska.

Our goal for this study was to analyze the relationship between sea otter diet and prey nutritional composition. Our objectives were to (1) investigate the macronutrient quality of sea otter prey in southern Southeast Alaska across seasons and (2) examine diet composition according to prey macronutrient composition across different sea otter metrics: (i) time since recolonization, (ii) sea otter sex and reproductive class, and (iii) season. This work is valuable to the understanding of sea otter prey macronutrient composition in Southeast Alaska. Understanding the prey composition gives us insight into sea otter dietary needs across seasons. This increases the potential to predict future impacts of sea otters on nearshore ecosystems, as sea otters recolonize Southeast Alaska and reduce the availability of shellfish in commercial and subsistence fisheries.

## 2 METHODS

### 2.1 Visual foraging observations

**FIGURE 1:**
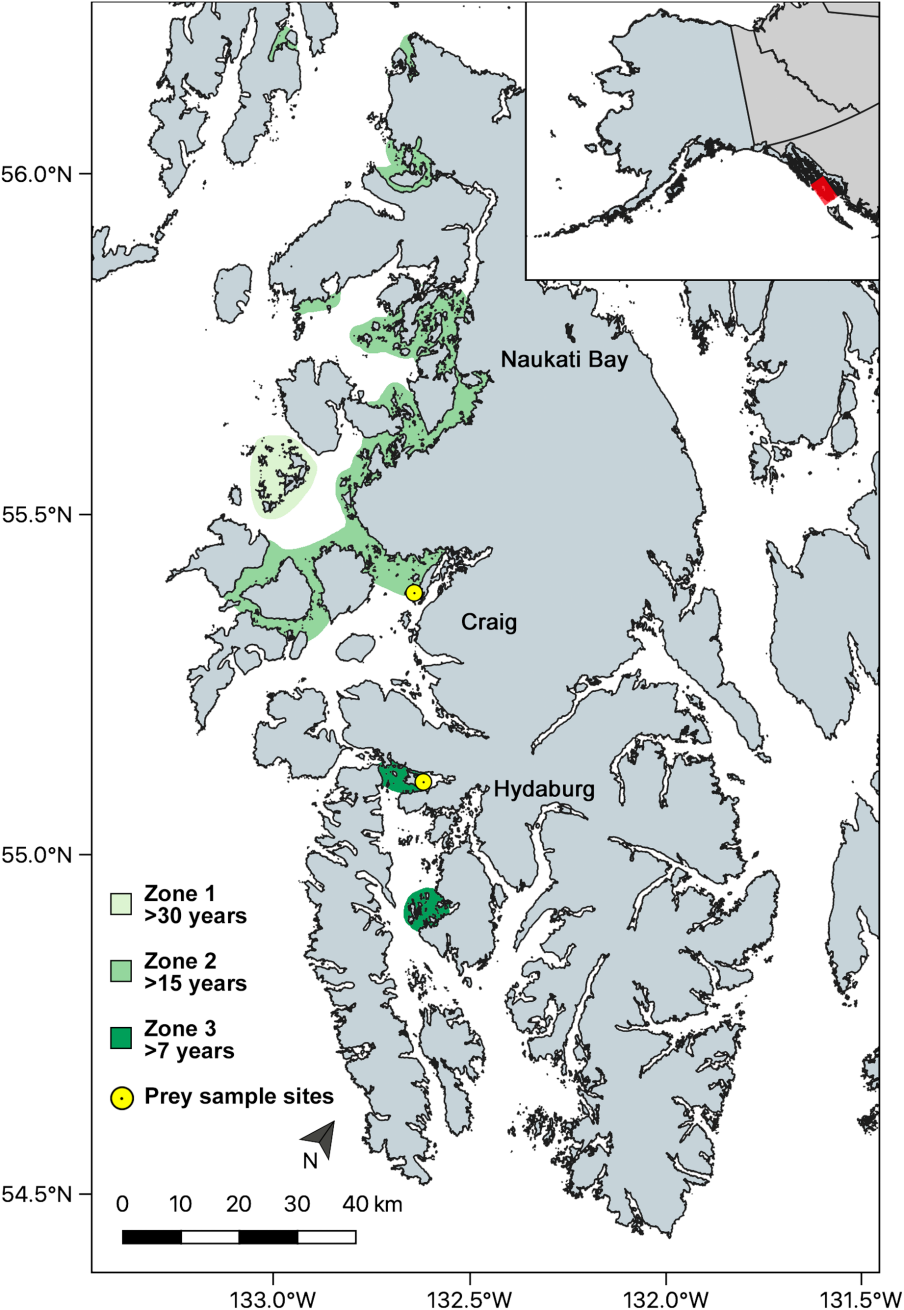
Sea otter visual foraging observations were made within three foraging zones (shaded areas with zone numbers listed) on Prince of Wales Island in southern Southeast Alaska. Each zone was designated by time‐since‐recolonization based on US Fish and Wildlife Service aerial surveys. Prey sample sites (1, Craig and 2, Soda Bay, near Hydaburg) are in yellow.

We observed sea otter foraging behavior from May to August 2018 on the western side and neighboring islands of POW. Sampling was stratified by time since recolonization, based on US Fish and Wildlife Service (USFWS) aerial surveys. Three periods were denoted from the surveys: zone 1 (>30 years present), zone 2 (≤30 years and ≥15 years present), and zone 3 (<15 years and >7 years present) (Figure 1). In each zone, a minimum of 50 forage bouts were recorded. Because zone 2 makes up a majority of POW, most foraging observations occurred in this zone.

Foraging observations were made from shore to assess sea otter diet composition. Questar telescopes (50–80X) were used to follow individual sea otters for one foraging bout (up to 20 dives per sea otter). The observer recorded the following foraging metrics: prey item (to species level when possible), prey size (based on an estimated sea otter paw width of 5.2 cm and categorized into <⅓ of the paw, >⅓ to <⅔ of the paw, or > ⅔ to the whole paw for items less than one paw and up to four paw lengths; anything over four paw lengths is estimated in cm), the proportion of the prey items consumed, GPS location (approximated based on GPS location of the telescope and distance/bearing to the sea otter), prey handling time (defined as the amount of time the sea otter spent manipulating and eating the prey), time spent diving, and total time spent at the surface (Coletti et al., 2016; Dean et al., 2002; Tinker et al., 2008). The following sea otter metrics were also recorded for each foraging bout: sex, reproductive status, and age class. Males were identified by the presence of a penile bulge, whereas females were identified if there was a clear lack of penile bulge, or if they had a pup. If sex was not confirmed, nor pup was observed, the sex was categorized as “unknown.” When possible, age class was determined as adult or juvenile by visual assessment of size and amount of grizzled fur (Lee et al., 2010).

We calculated the energetic intake rate (EIR) for sea otters based on visual foraging observations using the Sea Otter Foraging Analysis (SOFA) program, which is based in Matlab (MathWorks) and is a Bayesian model that uses a Monte Carlo‐based simulation to account for unknown prey items and potential sampling bias (Tinker et al., 2008). Prey diversity for each sea otter metric was calculated using the Shannon‐Wiener Index (Shannon, 1948). Success rate, which is defined as the percentage of dives in a bout where the sea otter came up with food, was calculated for each sea otter metric.

### 2.2 Prey sampling

Potential prey items to be collected for macronutrient and energetic analyses were selected based on existing literature on sea otter diets in Southeast Alaska (Hoyt, 2015; Weitzman, 2013). Five functional prey groups (crabs, clams, sea cucumbers, snails, and sea urchins) were identified that were composed of 13 target species for analysis. These five functional groups made up 95% of sea otter diets (in terms of biomass) from our visual foraging observations during this study. Five individuals of each target species were collected at two sites (Figure 1) in May 2018, August 2018, and February 2019. All samples were collected in the intertidal zone. Two collection sites were selected that encompassed the foraging observations across the POW region and had reliable access (Figure 1). Craig (Site 1) and Soda Bay (Site 2) represented differences in sea otter occupation time (>15 years for Craig, and >7 years for Soda Bay). Where there were more sea otters present, we had more difficulty locating invertebrate prey. Additional samples were opportunistically collected around POW if they were not present or in high enough abundance in the two designated sites. Samples were held in seawater‐filled buckets, cleaned of sand and dirt, and then frozen at −18°C.

In the lab, samples were thawed, weighed, measured, and separated into edible and inedible tissues. For bivalves, decapods, gastropods, and sea urchins, all hard parts were removed, weighed, and discarded, as they were considered inedible as the sea otter excretes these contents (Kenyon, 1969). For sea cucumbers, the entire organism was considered edible. Only crabs were processed separately by sex to determine differences between gravid females vs. non‐gravid females and males. The remaining edible tissues were weighed and homogenized in a Cuisinart Mini‐prep food processor. A maximum of 4 g of tissue was dried in a LECO Thermogravimetric Analyzer 701 (TGA) dryer at 135°C, or in a gravity convection oven (VWR Symphony 414004‐552) at 70°C. Standards and duplicates were run with each dryer to confirm consistent moisture values.

### 2.3 Energy content nutritional analysis

We measured energy content and proximate composition (the proportion of protein content, lipid content, moisture, and percent ash) for sea otter prey items. We used previously established methods (Fergusson et al., 2010) to measure energy density using a Parr 6725 semi‐micro bomb calorimeter. Standards and replicates were used to confirm consistent calorimeter readings. Lipid content was determined using previously established methods (Fergusson et al., 2020) using a sulfo‐phospho‐vanillin colorimetric analysis. Protein content was estimated by multiplying total nitrogen content by 6.25, which accounts for the nitrogen content of protein (Craig et al., 1978). Nitrogen content was measured with a FlashSmart elemental analyzer coupled to a Delta‐V continuous‐flow isotope ratio mass spectrometer (Thermo Scientific, Waltham, Massachusetts, USA). Carbohydrate content was not assessed as it is assumed to be negligible in marine invertebrates (Oftedal et al., 2007; Vollenweider et al., 2011). Ash content was processed at 600°C and measured with a LECO Thermogravimetric Analyzer 701 (TGA) dryer. Only samples with >1 g of dried material were able to be combusted for percent ash content.

### 2.4 Statistical analysis of sea otter prey

To test our first objective (investigate the macronutrient quality of sea otter prey across seasons), we calculated the percent protein and lipid of each prey group using the energy equivalents of 9.5 kcal/g for lipid and 5.7 kcal/g for protein (Kleiber, 1961). We used PRIMER v7 (Clarke & Gorley, 2015) with a one‐way analysis of similarity (ANOSIM) procedure with season as factors for each prey group, excluding crabs (*α* = 0.05). Snails had too few samples to conduct pairwise seasonal comparisons. Mussels were excluded from the results because they were a very small portion (<1%) of the sea otter diet around POW. Crabs were analyzed separately with season and sex as factors, using a two‐way ANOSIM (*α* = 0.05).

To test our second objective (compare diet composition and prey macronutrient composition across different sea otter metrics), we calculated regional‐level concentrations of macronutrient composition of sea otter diets using established methods (Oftedal et al., 2007). In brief, first, we converted the dry mass average (kcal, lipid, and protein) for each functional prey group to a wet mass value. Second, using the proportion of diet (also in wet mass) from SOFA outputs for each functional prey group and the average prey value (kcal, lipid, and protein), we calculated an average for each prey and macronutrient and added all individual groups together. Finally, we divided this newly calculated wet mass by dry mass to get the nutrient composition of dry mass. Statistical comparisons between sea otter metrics tested were not possible because we were not able to study known individual sea otters and all data were used at a regional scale including Western POW and surrounding islands (Oftedal et al., 2007).

Due to weather and light limitations, it was only possible to conduct visual foraging observations during spring and summer. To estimate year‐round diets, we used results from stable isotope analysis (LaRoche et al., 2021). Diet estimates were made using stable isotope (δ^13^C and δ^15^N) analysis of both sea otter vibrissae and the prey present around POW using a Bayesian model to estimate percent of total diet for functional prey groups. These diet estimates were used in the calculations for seasonal sea otter macronutrient contribution and comparison to changes in sea otter prey. All datasets are archived in a publicly accessible database with the Knowledge Network for Biocomplexity (LaRoche, Fergusson, & Pearson, 2020; LaRoche, King, & Pearson, 2020).

## 3 RESULTS

### 3.1 Sea otter diets across Prince of Wales Island

Foraging records consisted of 362 foraging bouts. In total, 3523 dives were recorded between May 6, 2018, and August 13, 2018. The overall foraging success rate was 89.9%, and diet diversity (*H*′) was 0.81. Mean ± SD dive time was 88.4 ± 44.5 s and mean ± SD surface time was 56.4 ± 42.3 s. The mean ± SD energy intake rate was 7.3 ± 0.22 Kcal/min. Sea otters were observed to consume a total of 44 prey types. When prey selection was examined irrespective of habitat type or site, the dominant prey categories (making up 97.5% of the total diet by biomass) calculated by SOFA were clams (80.9 ± 2.21%), sea cucumbers (8.5 ± 1.13%), crabs (3.8 ± 0.44%), snails (2.7 ± 0.39%), and sea urchins (1.6 ± 0.28%). Within the clam category, butter clams (*Saxidomus gigantea*) were the predominant species identified, comprising 36.9 ± 1.61% of the overall diet.

**FIGURE 2:**
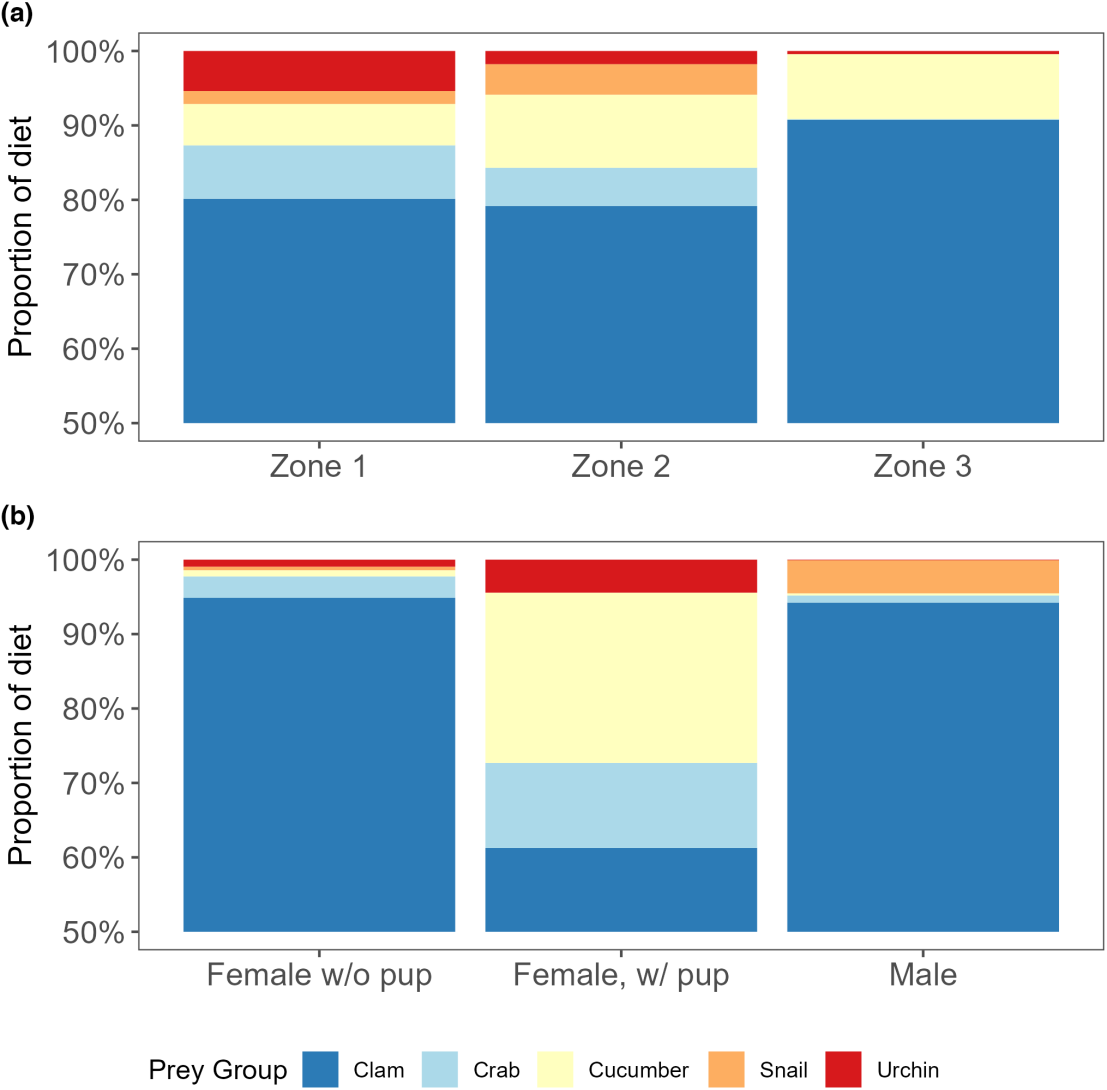
Proportion of diet from biomass estimates for time‐since‐recolonization zones (a) and sea otter reproductive status/sex (b). Zones are based on US Fish and Wildlife Service aerial surveys. Zone 1 is the area occupied for >30 years, Zone 2 is the area occupied >15 years, and Zone 3 is the area occupied >7 years.

We observed differences in sea otter diets across recolonization zones and reproductive status. Species diversity and intake rate (kcal/min) varied by recolonization zone (Table 1). The newest recolonization zone (>7 years) had the lowest species diversity (*H*′ = 0.39) and highest intake rate (11.06 ± 0.8 Kcal/min). The zone where sea otters have been present for the longest period (>30 years) had the highest species diversity (*H*′ = 1.19) and lowest intake rate (5.7 ± 0.8 Kcal/min). Clams were the main prey consumed across all recolonization zones (Figure 2a). Species diversity and intake rate varied by reproductive status (Table 1). Although clams were the dominant prey, sea otter diets varied by reproductive status; females with pups had a more varied diet and higher species diversity than females without pups and males (Table 1, Figure 2b). Females with pups had the highest species diversity (*H*′ = 1.23) and highest success rate (90%) whereas males had the lowest species diversity (*H*′ = 0.34).

**TABLE 1:** Sea otter intake rates (in kilocalorie per minute of foraging), species diversity (*H*′, Shannon Weiner Index), and dive statistics by recolonization zone and reproductive status/sex.

	*n* (bouts)	*n* (dives)	Kcal/min intake rate	SD	*H*′	Mean dive time	SD	Mean surface time	SD	Success rate
*Recolonization zones (number of years occupied)*										
Zone 1 (>30 years)	34	305	5.7	0.8	1.19	104.6	46.2	58.4	39.7	90.2%
Zone 2 (>15 years)	270	2864	7.0	0.4	0.90	83.7	43.6	52.2	40.0	89.6%
Zone 3 (>7 years)	58	354	11.6	0.8	0.39	101.9	41.6	85.2	50.6	90.9%
*Reproductive status/sex*										
Female no pup	37	484	7.0	0.7	0.44	84.1	43.3	50.7	35.6	90.1%
Female with pup	75	849	6.0	0.4	1.23	80.9	45.3	53.3	38.9	93.0%
Male	69	821	7.1	0.5	0.34	87.4	40.7	58.9	45.4	85.7%

### 3.2 Macronutrient content in sea otter diets

**FIGURE 3:**
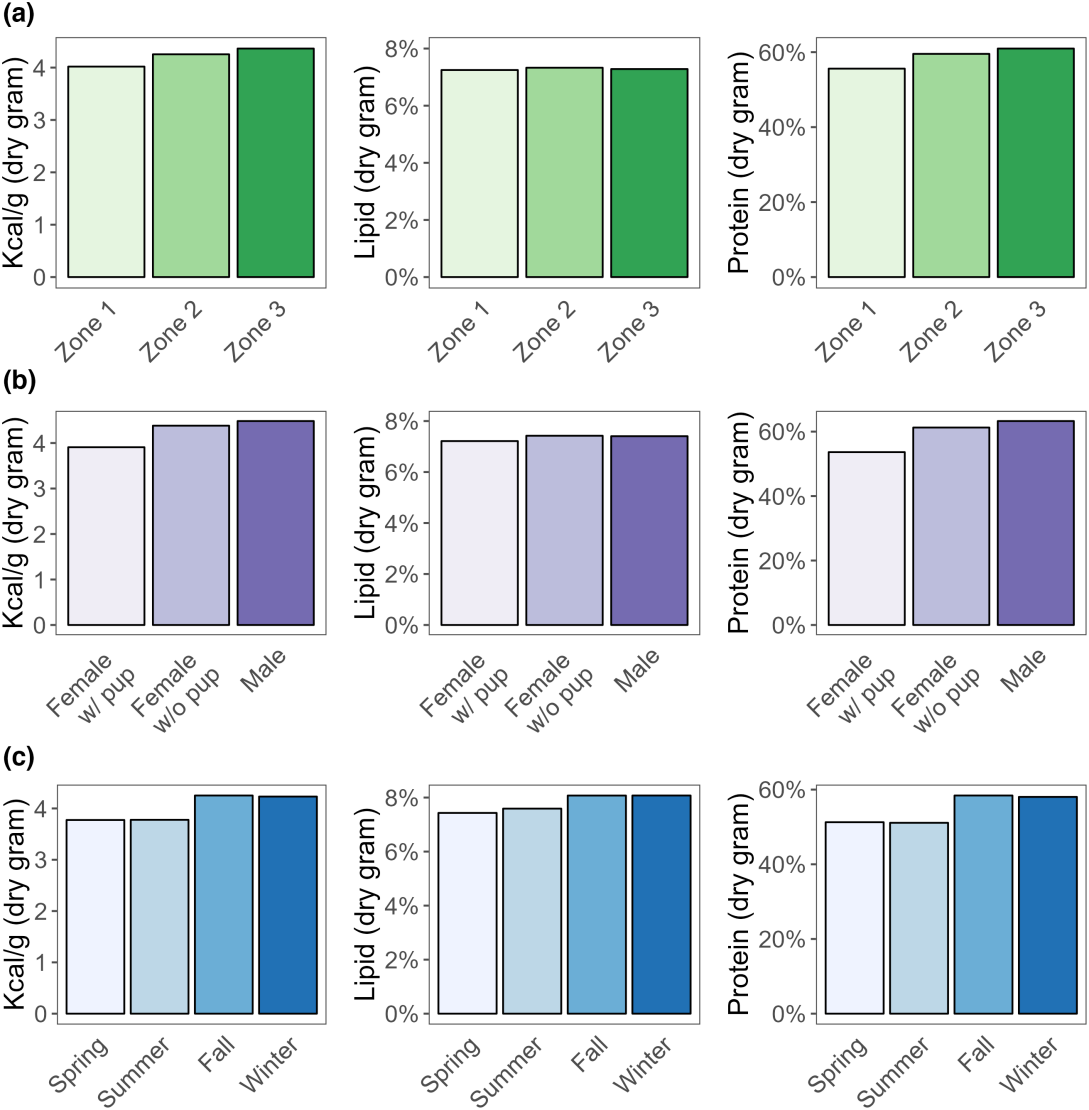
Comparison of the energy, lipid, and protein concentrations in population‐level diets for time‐since‐recolonization zone (a), sea otter reproductive status/sex (b), and season (c). All calculations are made from the wet mass (as a sea otter would eat the item) and converted to dry mass for comparison. Zones are based on US Fish and Wildlife Service aerial surveys. Zone 1 is the area occupied for >30 years, Zone 2 is the area occupied ≤30 and ≥15 years, and Zone 3 is the area occupied <15 and >7 years.

Energy content (kcal/g) and percent protein composition varied by recolonization zone (Figure 3a) and reproductive status (Figure 3b). Energy content, percent lipid, and protein composition appeared to vary by season (Figure 3d). For example, fall and winter varied from spring and summer in overall energy content (fall: 4.3 kcal/g, winter: 4.2 kcal/g, spring: 3.8 kcal/g, summer: 3.8 kcal/g). The variation in energy content is driven by both percent protein and percent lipid (fall: 8.0% lipid and 58.4% protein, winter: 8.1% lipid and 58.0% protein, spring: 7.4% lipid and 51.3% protein, summer: 7.5% lipid and 51.1% protein). In contrast, females with and without pups had variation in overall energy content driven only by the percent protein in the diet (with pup: 3.9 kcal/g, 7.2% lipid, and 53.6% protein, without pup: 4.4 kcal/g, 7.4% lipid, and 61.2% protein).

### 3.3 Energy content of sea otter prey

**FIGURE 4:**
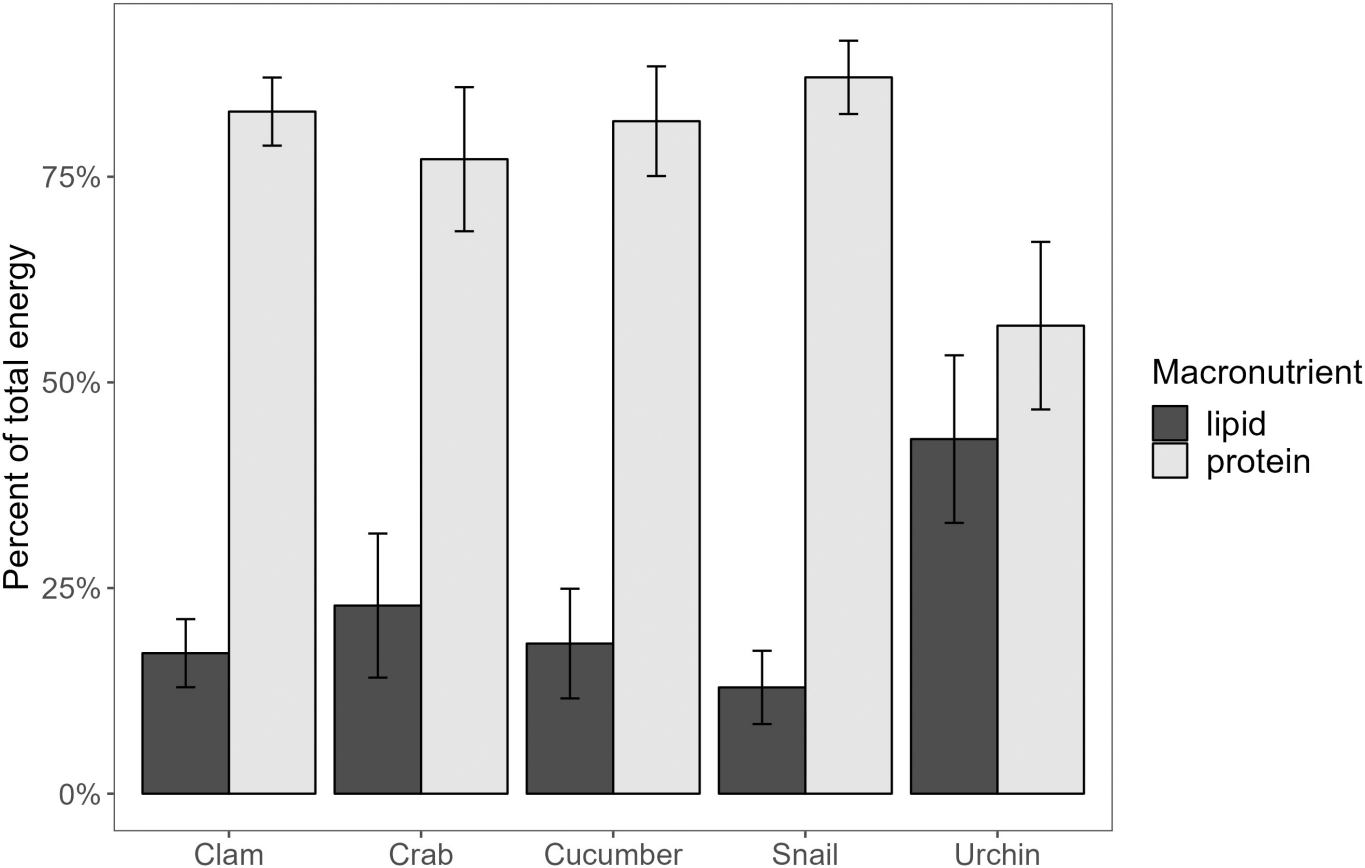
Mean (±SE) proportion of energy in each functional prey group derived from lipid and protein.

**FIGURE 5:**
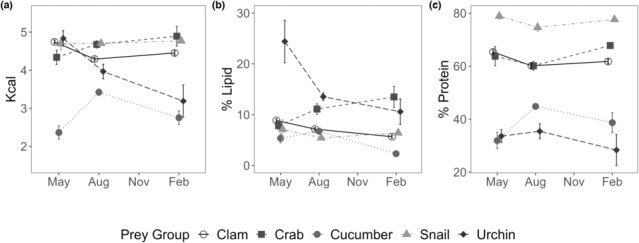
Seasonal whole‐body nutrition of functional prey groups of sea otters in dry mass: (a) mean (±SE) energy content in kilocalories per dry gram; (b) mean (±SE) % lipid content in dry grams; (c) mean (±SE) % protein content in dry grams.

For all functional prey groups except sea urchins, prey was protein‐rich, with low lipid content (Figure 4). Sea urchins were the only prey group significantly different from other prey groups for lipid‐to‐protein ratio (Figure 4, *p* < .01). Functional prey groups varied in their energy, lipid, and protein content across seasons (Table 2). Across all seasons, sea cucumbers exhibited lower energy than all other prey types (Figure 5), and their energy and lipid varied significantly by season (Table 2). Sea urchins had significant variability in lipid content across seasons (Table 2). Clams exhibited a significant change in energy and lipid over seasons as well, but *R* values were low, which means the overall seasonal effect was low (Table 2). Crabs did not vary significantly across seasons or sex. Snails did not vary significantly across seasons. Pairwise comparisons for all prey groups and seasons revealed significant differences in energy for clams and sea cucumbers (Table 3). Lipid varied for clams, sea cucumbers, and sea urchins, whereas protein only varied between seasons for clams. Snails were not compared across seasons due to the small sample size.

**TABLE 2:** Statistical comparison of sea otter prey groups with (A) season and (B) sex (for crabs only) as factors using analysis of similarity (ANOSIM). The *R* statistic ranges from near 0 (no difference between groups) and 1 (differences between groups) with bold numbers denoting significance (*p* < .05).

Functional prey group	Energy *R* statistic	*p* Value	Lipid *R* statistic	*p* Value	Protein *R* statistic	*p* Value
*A. Season*						
Clam	**.300**	.0001	**.213**	.0001	**.058**	.0160
Crab	.077	.1290	.024	.3250	**.133**	.0380
Sea Cucumber	**.472**	.0040	**.778**	.0010	**.267**	.0300
Sea Urchin	**.172**	.0430	**.404**	.0060	.067	.1900
Snail	−.157	.7300	−.158	.7460	.096	.1930
*B. Sex*						
Crab	.057	.2910	.065	.2630	**.181**	.0770

**TABLE 3:** Statistical pairwise comparisons of sea otter prey groups with season and sex (for crabs only) using analysis of similarity (ANOSIM). The *R* statistic ranges from near 0 (no difference between groups) and 1 (differences between groups) with bold numbers denoting significance (*p* < .05).

Functional prey group	Season		Energy R statistic	*p* Value	Lipid *R* statistic	*p* Value	Protein *R* statistic	*p* Value
Clam	Spring	Summer	**.470**	.000	**.082**	.036	**.125**	.000
	Spring	Winter	**.228**	.000	**.397**	.000	.034	.152
	Summer	Winter	**.117**	.001	**.117**	.016	−.018	.668
Crab	Spring	Summer	.021	.317	.069	.129	**.155**	.022
	Spring	Winter	.073	.328	−.102	.641	−.039	.477
	Summer	Winter	.150	.124	−.151	.763	.070	.244
Sea Cucumber	Spring	Summer	**.824**	.001	**.536**	.016	**.624**	.016
	Spring	Winter	.068	.238	**.672**	.008	.016	.389
	Summer	Winter	**.472**	.016	**1.000**	.008	.156	.103
Sea Urchin	Spring	Summer	.133	.073	**.426**	.019	−.089	.771
	Spring	Winter	**.380**	.024	.270	.064	.052	.226
	Summer	Winter	.140	.129	**.410**	.002	**.205**	.047

**FIGURE 6:**
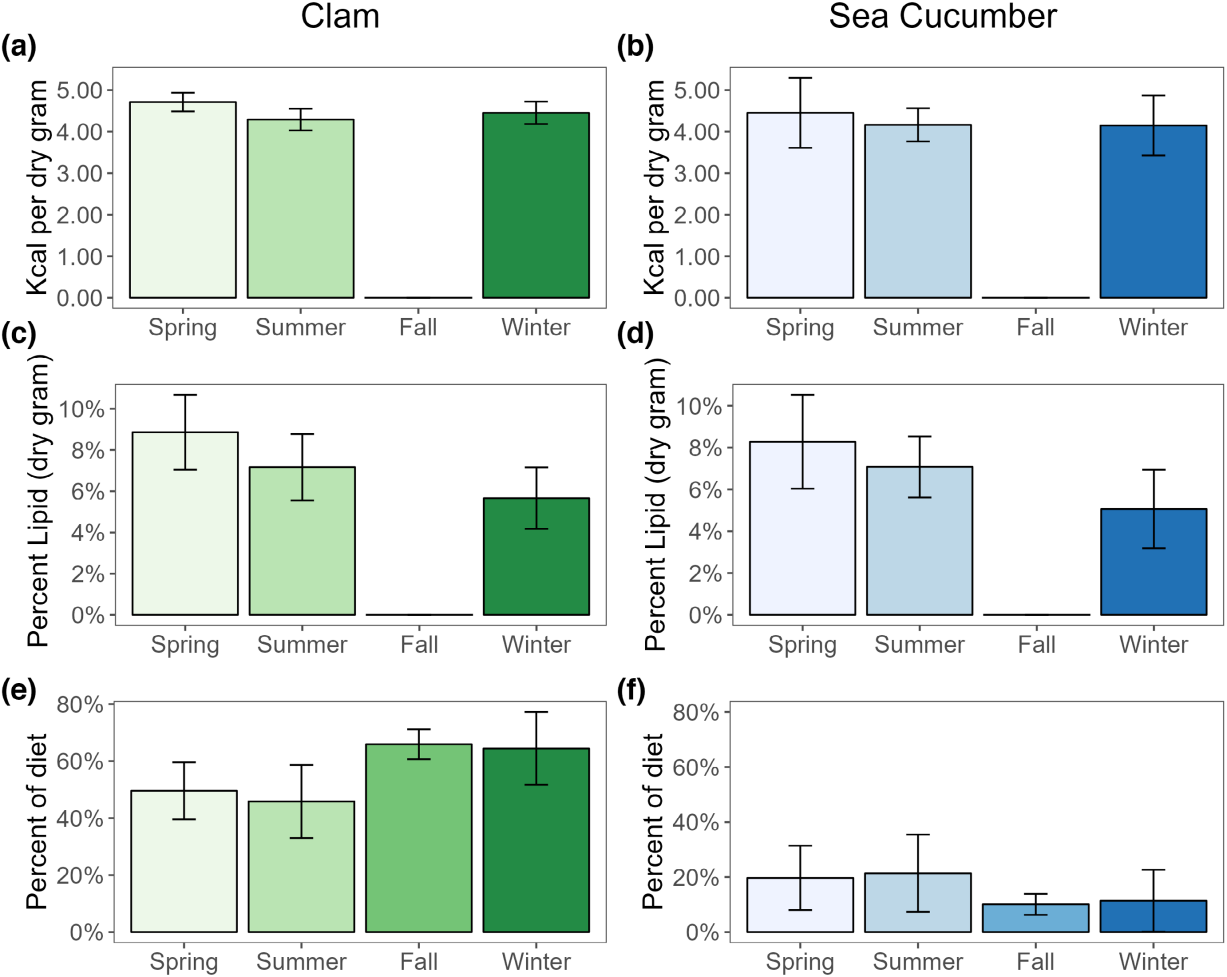
Seasonal variation in the nutrition of sea otter prey (green bars are clams, and blue bars are sea cucumbers. Error bars are standard deviation) and frequency of occurrence of that prey species in the diet (e, f, from LaRoche et al., 2021). a and b are energy density (kilocalorie per dry gram), and c and d are lipid content (percent per dry gram). Prey samples were not collected in Fall.

When we compared year‐round energetic changes in sea otter prey with diet proportion estimates, we found EIR of most functional prey groups did not correlate with energy density. Based on LaRoche et al. (2021), sea otters consumed more clams in the fall and winter months (Figure 6e), when the energy and lipid contents of clams were lower than the spring season which did not correspond with energy changes (Figure 6a) or lipid (Figure 6c). Sea cucumber consumption (Figure 6f), the second most abundant diet contribution (LaRoche et al., 2021), did appear to correspond with changes in energy (Figure 6b) and lipid (Figure 6d). Sea otters decreased their consumption of sea cucumber in the fall and winter months, which corresponded with declining energy content.

## 4 DISCUSSION

The main sea otter diet component regardless of location, sex, age, and season was clams. Sea otter intake rates in the POW region (EIR 7.3 kcal/min) were comparable to studies of sea otters at or near carrying capacity (Rechsteiner et al., 2019; Tinker et al., 2008, 2021), showing that at a regional level, sea otters of POW may be reaching carrying capacity. There were differences in the proportion of diet and prey composition according to time since recolonization, reproductive status, and season (Hoyt, 2015; Kvitek et al., 1993).

### 4.1 Time since recolonization

Time since recolonization affects the prey composition of sea otter diets. Although clams comprise the majority of the diet in all regions of POW, there were differences in prey composition across recolonization zones. Sea otter diets in areas of POW that have been colonized for the least amount of time (zone 3, colonized for >7 and < 15 years) had the lowest species diversity in prey and highest energy recovery rates. In this zone, clams were overwhelmingly present in the diets of sea otters. This result was similar to previous studies in mixed sediment communities in Southeast Alaska, where sea otters focus on fewer, high‐quality prey species (e.g., sea urchins in rocky habitats, large clams in soft‐sediment habitats) in newly occupied areas, and eventually diversify prey species as sea otter populations persist and preferred prey decline in size and abundance (Hoyt, 2015; Weitzman, 2013). Lipid content in sea otter diets was consistent across recolonization zones, but overall EIR was slightly higher in zone 3 which was colonized for >7 and <15 years (11.6 kcal/min), which may be due to the ability to obtain larger prey in newly colonized areas. Foraging records for the areas where sea otters had been present the longest (zone 1, colonized for >30 years) had an EIR (5.7 kcal/min) that was comparable to previous studies where sea otters were at carrying capacity (Coletti et al., 2016; Rechsteiner et al., 2019; Tinker et al., 2008, 2021). This shows that the sea otters in zone 1 of POW are likely at or near carrying capacity, which is in line with modeling from aerial survey data from the region (Eisaguirre et al., 2021; Tinker, Gill, et al., 2019).

When considering sea otter diets and how sea otter invertebrate removal can affect the nearshore system, it is important to look at diet variation across recolonization zones as diets in the newly colonized areas were less diverse, which is similar to past studies in Alaska (Hoyt, 2015). In the present study, the areas we observed where sea otters are newly colonizing were soft‐sediment habitats ripe with large butter clams, which were the overwhelmingly predominant prey item.

### 4.2 Reproductive status

Female sea otters with pups had a more varied diet composition than females without pups and males. The difference in the diet was the largest shift among all tested metrics. Females with pups ate a higher proportion of crabs, sea cucumbers, and sea urchins than sea otters without pups. Sea cucumbers are the functional prey group with the lowest energy content; therefore, females with pups are obtaining fewer calories per gram of food consumed. Success rates were high across the POW region, but females with pups had the highest success rate. One possibility could be that a female with a pup would rather come up with prey every dive instead of risking no success for a higher effort prey (such as choosing a slow‐moving sea cucumber laying the ocean floor, instead of digging for a clam or a fast‐moving crab). Other studies have reported females to vary their diet according to their reproductive state. In California, tagged female sea otters switched their foraging strategies and prey types consumed when they had no pup, small pups, and large pups (Staedler, 2011).

There were other sex‐specific differences in prey type. Males were observed to eat more snails than females, while females with pups were never observed to eat snails. In previous studies, snail specialists were linked to poor overall body condition and higher death rates due to disease (Tinker, Tomoleoni, et al., 2019). Because we did not follow individuals in this study, we were unable to distinguish a similar pattern in Southeast Alaska sea otters. In addition, although the prevalence of geoduck clams (*Panopea generosa*) in diets was low in our study, with only seven observations of geoduck clams consumed throughout the region, all of these were consumed by males. Geoduck clams are higher risk prey because of the increased effort needed to excavate them; thus, sea otters generally make several dives to recover one geoduck clam (Kvitek et al., 1993). We did not observe sea otters foraging for geoduck clams in the most recently colonized zones, which matches previous studies in the same region (Hoyt, 2015). These areas may have larger clams that reside in shallower areas (e.g., butter clams) creating a more energetically efficient option. Dietary differences between males and females are worth noting, as males are more likely to expand into new regions first (Garshelis & Garshelis, 1984; Lafferty & Tinker, 2014). As new regions are being recolonized, knowing the diet preferences of males can help to predict invertebrate predation in relation to species that are of interest to humans.

Lipid content and energy content were lowest in the diets of females with pups. There were no significant differences in dive or surface times for females with pups vs. other age/sex classes. However, females with pups have the highest foraging success rate when compared to females without pups and males, which could indicate that females with pups select less energy‐rich prey items over the risk of no success. Previous studies of sea otter energetics showed that female sea otters with large pups operate at an energetic deficit by the time a pup reaches weaning age (Chinn et al., 2016; Thometz et al., 2016). In the present study, this deficit may be evidenced by the lower caloric content of prey consumed and lower overall EIR for females with pups. Most forage bouts of females with pups in this study came from zone 2 (otters present 7–30 years), so we were unable to determine if the lower EIR is due to a bias in females without pups and males from the higher EIR zone 3 (<7 years) region.

### 4.3 Seasonal diet shifts

Sea otters consume prey with higher percent lipid in the fall and winter months. This change in nutrient composition could be due to prey switching due to availability or to compensate for colder temperatures in winter months and the need to obtain more lipids to metabolize for warmth. Average sea surface temperatures (SST) for nearby Ketchikan range from a low of 6°C in the winter to a high of 14°C in the summer (NCEI Coastal Water Temperature Guide—Alaska Coast Table, n.d.). The low end of the water temperature range in which a sea otter can remain in a thermoneutral zone (i.e., a physiological state whereby the animal maintains its normal core body temperature without metabolic heat production or active cooling) is about 15°C (Davis, 2019; Murray, 2015). This temperature is slightly above the typical summer SST on POW, meaning that at temperatures below this critical level, sea otters must consume more energy to generate additional heat. Previous studies have shown that sea otters forage for longer periods in winter months (Esslinger et al., 2014), as well as spending more time hauled out (Gelatt et al., 2002). Although sea otters do not undergo a full molt like many marine mammals, Kenyon (1969) reports a study of fur molt in captive animals, showing that more fur was present in the drains in spring and summer months. Currently, to our knowledge, there are no published studies that test the density and characteristics of sea otter fur across seasons in Southeast Alaska, so it is unknown if the fur is thicker and can add additional warmth in the winter months.

Based on sea otter diet estimates from LaRoche et al. (2021), clam consumption in spring and summer was lower than in fall and winter. This change, which does not correlate with seasonal changes in energy or lipid content, could be due to paralytic shellfish poisoning (PSP). PSP is a toxin in algae that blooms in the spring and summer months along the Pacific coast (Kvitek et al., 1991). Studies have shown that sea otters will still eat bivalves that have PSP toxins present, but will avoid bivalves with very high amounts, as well as only consuming the foot and discarding the siphon, which usually has the highest concentration of PSP (Kvitek et al., 1991).

Sea otter consumption of sea cucumbers across seasons positively corresponded with sea cucumber total energy and lipid content (Figure 6). Sea cucumbers are broadcast spawners. They move into shallow waters in the late spring to begin spawning in the summer months (Cameron & Fankboner, 1986). During the fall and winter months, they retreat to deeper water. Their highest percent lipid and caloric content were observed in the summer when they are preparing to spawn. Estimates from stable isotope analyses show the highest diet proportion in the summer, with spring slightly lower, and a drastic drop in the fall and winter months (LaRoche et al., 2021). This correlates with the sea cucumber's life history. The visual foraging observations show higher consumption in the summer than in the spring. This correlation can be due to increased caloric content. Additionally, their shallow‐water summer habitat makes them more easily obtainable for sea otters. At other times of the year, sea cucumbers inhabit depths up to 250 m, which is outside of a sea otter's diving ability (Bodkin et al., 2004; Cameron & Fankboner, 1986; Costa & Kooyman, 1982).

### 4.4 Caveats, management implications, and future work

One limitation of this study is that all forage data were collected in 1 year, which could lead to a bias in a potentially anomalous year. Past studies on sea otter foraging in this region (Hoyt, 2015; Kvitek et al., 1993), show similar and consistent sea otter diet patterns across time and reinforce the broad utility of our results.

It is important for managers to consider the whole ecosystem and not only focus on each single species individually. Because sea otters are removing biomass similar to a fishery, their impact on invertebrate prey should be considered in management strategies. It is also important to incorporate how sea otters prioritize and change their diets; for example, this study showed that sea otters increase their sea cucumber consumption in the summer months. Currently, there is a lack of invertebrate surveys to assess current population levels. Dungeness crabs, butter clams, and other subsistence bivalves like cockles are not surveyed. Ecosystem‐level management for the Alaska nearshore system with a specific interest in subsistence foods would account for the needs of people both local and commercial fishing, as well as the health of the ecosystem.

Within our study area, only three aerial surveys were conducted over 30 years to estimate the sea otter abundance and geographic range (Tinker, Gill, et al., 2019; USFWS, 2014). More fine‐scale outcomes of sea otter diet changes were not possible because of the long gaps in population data. More frequent surveys are needed to better understand the population size and distribution. There was an 11‐year gap between the current Southeast Alaska population surveys and the one previous (Eisaguirre et al., 2021; Schutte et al., 2023; Tinker, Gill, et al., 2019). Because of the cost‐prohibited nature of large‐scale survey efforts, more frequent surveys on regional levels may be the most efficient route to monitor the changing sea otter distribution. Community‐driven boat‐based surveys that focus on subsistence use areas could be one effective method.

## 5 CONCLUSIONS

Sea otters can be used as a looking glass into the overall ecosystem due to their foraging habits. Sea otters sample benthic invertebrates at a higher rate and with better skill than people can attain with SCUBA surveys (Oftedal et al., 2007; Riedman & Estes, 1990). This study provides quantitative data that can inform an ecosystem‐based management approach that also considers local subsistence harvest needs and stakeholder input. However, for a more comprehensive understanding of the sea otter population in Southeast Alaska, abundance surveys of both sea otters and invertebrates need to be conducted more frequently. If used in conjunction with more comprehensive abundance surveys, the quantitative results presented here regarding sex and location‐specific diet composition can be used to predict current and future sea otter ecosystem effects, thus informing co‐management plans for this apex predator in addition to commercially important and subsistence foods.

## AUTHOR CONTRIBUTIONS

**Nicole LaRoche:** Conceptualization (equal); data curation (lead); formal analysis (lead); funding acquisition (supporting); investigation (lead); methodology (equal); project administration (equal); resources (equal); software (equal); validation (equal); visualization (equal); writing – original draft (lead); writing – review and editing (equal). **Sydney King:** Data curation (supporting); investigation (supporting); resources (supporting); validation (supporting); writing – review and editing (supporting). **Emily Fergusson:** Data curation (supporting); methodology (supporting); software (supporting); validation (supporting); visualization (supporting); writing – review and editing (supporting). **Ginny Eckert:** Conceptualization (equal); data curation (supporting); formal analysis (supporting); funding acquisition (equal); investigation (supporting); methodology (supporting); project administration (supporting); resources (supporting); supervision (supporting); validation (supporting); writing – review and editing (supporting). **Heidi Pearson:** Conceptualization (equal); data curation (supporting); formal analysis (supporting); funding acquisition (equal); investigation (supporting); methodology (supporting); project administration (equal); supervision (equal); validation (supporting); visualization (supporting); writing – original draft (supporting); writing – review and editing (equal).

## CONFLICT OF INTEREST STATEMENT

None.

### OPEN RESEARCH BADGES

This article has earned an Open Data badge for making publicly available the digitally‐shareable data necessary to reproduce the reported results. The data is available at https://doi.org/10.5063/F1C53J8X; https://doi.org/10.5063/F1Q23XNG.

## Data Availability

Visual foraging observation data used in this study are published through KNB at: https://doi.org/10.5063/F1C53J8X (LaRoche, King, & Pearson, 2020). Invertebrate biomass data are published through KNB at: https://doi.org/10.5063/F1Q23XNG (LaRoche, Fergusson, & Pearson, 2020).
